# Update on an Observational, Clinically Useful Gait Coordination Measure: The Gait Assessment and Intervention Tool (G.A.I.T.)

**DOI:** 10.3390/brainsci12081104

**Published:** 2022-08-19

**Authors:** Janis J. Daly, Jessica P. McCabe, María Dolores Gor-García-Fogeda, Joan C. Nethery

**Affiliations:** 1 Brain Rehabilitation Research Center, Malcom Randall VA Medical Center, Gainesville, FL 32608, USA; 2 Department of Physical Therapy, College of Public Health and Health Professions, University of Florida, Gainesville, FL 32608, USA; 3 Department of Neurology, School of Medicine, Case Western Reserve University, Cleveland, OH 44108, USA; 4 Brain Plasticity and NeuroRecovery Laboratory, Cleveland VA Medical Center, Cleveland, OH 44106, USA; 5 Department of Physical Therapy, Occupational Therapy, Physical Medicine and Rehabilitation Faculty of Health Sciences, Rey Juan Carlos University, 28032 Madrid, Spain; 6 Cognitive and Motor Learning Program, Cleveland VA Medical Center, Cleveland, OH 44106, USA

**Keywords:** gait, coordination, central nervous system, motor control, observational gait scales, stroke, multiple sclerosis, MS

## Abstract

With discoveries of brain and spinal cord mechanisms that control gait, and disrupt gait coordination after disease or injury, and that respond to motor training for those with neurological disease or injury, there is greater ability to construct more efficacious gait coordination training paradigms. Therefore, it is critical in these contemporary times, to use the most precise, sensitive, homogeneous (i.e., domain-specific), and comprehensive measures available to assess gait coordination, dyscoordination, and changes in response to treatment. Gait coordination is defined as the simultaneous performance of the spatial and temporal components of gait. While kinematic gait measures are considered the gold standard, the equipment and analysis cost and time preclude their use in most clinics. At the same time, observational gait coordination scales can be considered. Two independent groups identified the Gait Assessment and Intervention Tool (G.A.I.T.) as the most suitable scale for both research and clinical practice, compared to other observational gait scales, since it has been proven to be valid, reliable, sensitive to change, homogeneous, and comprehensive. The G.A.I.T. has shown strong reliability, validity, and sensitive precision for those with stroke or multiple sclerosis (MS). The G.A.I.T. has been translated into four languages (English, Spanish, Taiwanese, and Portuguese (translation is complete, but not yet published)), and is in use in at least 10 countries. As a contribution to the field, and in view of the evidence for continued usefulness and international use for the G.A.I.T. measure, we have provided this update, as well as an open access copy of the measure for use in clinical practice and research, as well as directions for administering the G.A.I.T.

## 1. Gait Coordination Importance and Definition

There have been important discoveries of brain and spinal cord mechanisms controlling gait coordination (e.g., [1,2,3,4,5,6,7]). Additionally, there is growing evidence identifying the neural pathologies and resulting impairments underlying gait dyscoordination after stroke and other neurological diagnoses, and their response to treatment (e.g., [8,9,10,11,12]). For stroke, these include description not only of the disruption of neural control, but its manifestation in terms of gait dyscoordination, for example, abnormal co-contractions [13] and interference with the normal coordinated interactions of the mechanical energetics of the lower limbs [14].

Based on the available discoveries, there is a greater ability to construct more efficacious gait coordination training paradigms for those with neurological injury or disease. With new interventions targeted at improving the neural drive of gait coordination, it is critical in these contemporary times to use the most precise, sensitive, homogeneous (i.e., domain-specific [15]), and comprehensive measures available [16,17] to assess both gait coordination itself, as well as its underlying domains of strength, limb joint coordinated movement, coordination of muscle co-contractions, proprioception, and balance [18,19,20]. Indeed, others have called for and suggested a standardized set of assessment measures [21], but pertaining to gait coordination, this most recent suggestion was limited to only an incomplete gait measure [16,17].

Gait coordination is defined well by Krasovsky and Levin [22], page 213, as follows:

“Locomotor coordination is a context-dependent property of the motor system, having both spatial and temporal components. Spatial coordination is the relationship between the position of different body segments or joints, whereas temporal coordination is the relative timing between segment or joint positions throughout the task. These components are never mutually exclusive……”

Therefore, an example of a gait coordination measure is ‘knee flexion angle at toe-off’. In this example, the spatial component is knee flexion angle and the temporal component is the gait event time of toe-off. Without both the spatial and temporal components simultaneously being performed, there is no measure of coordination. The technology-based gold standard of gait coordination measurement and force production includes gait kinematics and gait kinetics, customarily measured using motion capture and force plate systems in well-equipped gait research laboratories [23]. Studies have shown that gait kinematics is an important quantitative measure of gait coordination and is associated with observed abnormal co-contractions after stroke, causing gait dyscoordination [24].

## 2. Observational Gait Coordination Scale with Precision, Sensitivity, Reliability, Validity, Homogeneity, and Comprehensiveness

In clinical practice and in some gait research settings, there is no access to such technology. Furthermore, the time constraints of clinical practice make it impractical to utilize this instrumented technology in its current form due to the labor-intensive process of collecting and analyzing the data. However, observational gait coordination measures can be used in that case. The ideal observational gait coordination measure would be constructed according to the following characteristics:

Homogenous, that is, all items measuring gait coordination/dyscoordination, according to the above definition of coordination, and no items assessing compensatory strategies.

As comprehensive as possible, that is, measuring as many coordination joint movements and other gait components as possible.

Numerical scoring scheme overall, and per item, with score numbers within an item assigned across levels of dyscoordination.

Good psychometrics (e.g., reliability, validity).

Good sensitivity in measuring the recovery of gait coordination, both endogenous and in response to treatment.

Practical, with a reasonable scoring time and the use of equipment available in most clinical settings.

Two separate research groups conducted a review of observational gait measures [16,17]. They concluded that the Gait Assessment and Intervention Tool (G.A.I.T.) was the most suitable scale for both research and clinical practice compared to other observational gait scales, since it has been proven to be valid, reliable, sensitive to change, homogeneous, and comprehensive (Table 1 and Table 2). Since that time, the developers of the G.A.I.T. have received requests and notices indicating that clinicians and researchers are using the G.A.I.T. in the following countries: Turkey, Slovenia, Italy (three separate users), Korea, India (two separate users), Australia (two separate users), Columbia, Spain, Thailand, and the U.S. Additionally, the G.A.I.T. has been translated into the following languages: Spanish [25], Taiwanese [26], and Portuguese (translation is complete, but not yet published).

## 3. Use of G.A.I.T. for Those with Stroke

The G.A.I.T. psychometrics were originally tested in stroke survivors. The G.A.I.T. was constructed with items measuring the spatial coordinated movement components of gait (movement excursion), that occur at specified temporal events of stance and swing phases of the gait cycle. Within each item of the measure, deviation from normal coordination is scored at multiple levels of dyscoordination. Psychometrics were studied and found to be good, according to intra-rater reliability (intraclass correlation (ICC) = 0.98; *p* = 0.0001; 95% confidence interval (CI)= 0.95–0.99), and inter-rater reliability (ICC = 0.83; *p* = 0.007; 95% CI = 0.32–0.96), including between an experienced and an inexperienced clinician (ICC = 0.996; *p* = 0.0001; 95% CI = 0.986–0.999) [27]. The G.A.I.T. showed measurement sensitivity in identifying a difference between two treatment groups in the recovery of gait coordination from pre- to post-treatment (that is, the G.A.I.T. showed that group 1 had a significant additive effect (parameter statistic = 1.10, *p* = 0.045, 95% CI= 0.023–2.18)) [28].

## 4. MCID

Very recently, two independent groups have estimated the minimal clinically important difference (MCID) for the G.A.I.T. in stroke survivors [29,30]. In a group of subacute patients (mean time since stroke was 45 days), a change in the G.A.I.T. score between 1.5 and 4 points was deemed the MCID value [29]. In a study of chronic stroke, the G.A.I.T. scores were well correlated to the Functional Ambulation Category (FAC) measure (r = 0.73; [30]. In that study, the G.A.I.T. was studied for MCID according to two anchors, the FAC and the speed-based functional categories of household, limited community, or full community ambulation [31]. They proposed the following G.A.I.T. MCID value: 11.8 points related to FAC (Functional Ambulation Category) level 3 or household ambulator gait classification; and 5.19 points related to FAC levels 4 and 5, or limited community ambulator, or full community ambulator classification [30]. In this particular study [30], the G.A.I.T. was scored bilaterally and averaged, which is a novel approach to the utilization of the measure; this procedure may have influenced the value of the MCID by diluting (averaging out) any improvement in the paretic limb and thus resulting in a higher calculated MCID. Given its advantageous characteristics [16,17], additional analyses to interpret the MCID value levels for meaningful change according to the G.A.I.T. are warranted; at the same time, these current studies are an important contribution to the field.

## 5. Use of the G.A.I.T. for Those with MS

Recently, the G.A.I.T. psychometrics were studied for those with multiple sclerosis (MS). Construct validity was tested [32]. The results showed a high construct validity, with correlations between the G.A.I.T. and the Rivermeade Gait Assessment (RVGA) at r > 0.90, and ranging in correlation with the Tinetti Gait Scale (TGS) from −0.62 to −0.59. We should note, in this regard, that in prior work, the authors of [17] compared the G.A.I.T. versus other gait measures such as the RVGA and TGS, and found the G.A.I.T. to be the most suitable scale for stroke survivors for both research and clinical practice compared to the other observational gait scales, since it has been proven to be valid, reliable, sensitive to change, homogeneous, and comprehensive. Further, for the MS population, they found that correlations were lower for scales that included speed, such as the Timed Up and Go Test. Reliability of the G.A.I.T. was high for use with those with MS (intraclass correlation coefficient for the intra-rater reliability, e.g., r = 0.91; 95% CI 0.85–0.95 for the right side; and inter-rater reliability, e.g., r = 0.91 (95% CI 0.85–0.95) for the right side) [33].

## 6. Contribution to the Field

In view of the evidence for the continued usefulness and international use for the G.A.I.T. measure, we have provided this update, as well as an open access copy of the measure for use in clinical practice and research (Table 1). We are also providing the prior published directions for administering the G.A.I.T., enhanced with additional information obtained from its continued use (Table 2).

## Figures and Tables

**Table 1:** Gait Assessment and Intervention Tool (G.A.I.T.)

Name____________________________________ Date_______________ Examiner_____________________	
Diagnosis___________________ Limb assessed_____ Device/Orthosis/Assist_________________________	
**Stance and Swing Phases**	
	** Score **
1. Shoulder position 0 = normal. 1 = abnormal position (check all that apply __ depressed, __ elevated, __ retracted, or __ protracted).	_____
2. Elbow flexion 0 = < 45° (normal = ~ 10°). 1 = 45 – 90° elbow flexion. 2 = > 90° elbow flexion.	_____
3. Arm swing 0 = normal. 1 = abnormal – reduced or absent arm swing.	_____
4. Trunk alignment (Static) 0 = normal erect posture (absence of flexion, extension or lateral flexion). 1 = trunk statically in __ flexion or __ extension. 2 = trunk statically in lateral flexion to the __ right or __ left. 3 = trunk in both __ flexion or __ extension, & lateral flexion to __ right or __ left.	_____
**Stance Phase**	
5. Trunk posture/movement (Dynamic) (sagittal plane) (lateral view) 0 = normal (static trunk alignment maintained). 1 = trunk __ flexes or __ extends (check one) < 30°. 2 = trunk __ flexes or __ extends (check one) 30° or more.	_____
6. Trunk posture/movement (Dynamic) (coronal plane) (front/back view) 0 = normal (static trunk alignment maintained). 1 = trunk laterally flexes to __ right or to __ left (check one) < 30°. 2 = trunk laterally flexes to __ right or to __ left (check one) 30° or more.	_____
7. Weight shift (lateral displacement of head, trunk and pelvis) (coronal plane) (front/back view) 0 = normal weight shift (~ 25 mm shift over stance limb). 1 = reduced weight shift. 2 = almost none or no weight shift. 2 = excessive weight shift.	_____
8. Pelvic position (coronal plane) (front/back view) 0 = normal (no Trendelenberg sign) 1 = mild pelvic drop on contralateral side. 2 = severe or abrupt pelvic drop on contralateral side.	_____
9. Hip extension (sagittal plane) (lateral view) 0 = normal (moves from 30° of hip flexion at initial contact to neutral by midstance, then to 20° of extension past neutral in terminal stance). 1 = hip extends to neutral by midstance but lacks further hip extension during terminal stance. 2 = abnormal throughout stance (hip remains in flexion or marked extension).	_____
10. Hip rotation (coronal plane) (front/back view) 0 = normal (remains in neutral) 1 = abnormal, internal rotation 1 = abnormal, external rotation	_____
11. Knee – initial contact phase (sagittal plane) (lateral view). Choose __ A or __ B (check selection) A. Knee flexion 0 = normal (knee in neutral/not hyperextended). 1 = 5° – 15° knee flexion. 2 = > 15°, but < 30° knee flexion. 3 = > 30° knee flexion. B. Knee extension 0 = normal (knee in neutral/not in flexion). 1 = 5° – 15° knee hyperextension. 2 = > 15° up to 30° knee hyperextension. 3 = > 30° knee hyperextension.	_____
12. Knee – loading response phase (sagittal plane) (lateral view). Choose __ A or __ B (check selection) A. Knee flexion 0 = normal (up to 15° knee flexion). 1 = > 15°, but < 30° knee flexion. 2 = ≥ 30° knee flexion B. Knee extension 0 = normal (up to 15° knee flexion). 1 = no knee flexion, up to 15° knee hyperextension. 2 = ≥ 15° knee hyperextension.	_____
13. Knee – midstance phase (sagittal plane) (lateral view). Choose __ A, __ B, __ C, or __ D (ck. select) A. Knee flexion 0 = normal (knee in 4° flexion at heel strike, increasing to 15° flexion at 14% of gait cycle). 1 = 5 – 15° flexion throughout midstance; does not achieve neutral at midstance. 2 = > 15°, but < 30° knee flexion 3 = ≥ 30° knee flexion. B. Knee extension 0 = normal (knee in 4° flexion at heel strike, increasing to 15° flexion at 14% of gait cycle). 1 = knee extended through midstance phase; not hyperextended. 2 = up to 15° knee hyperextension during midstance phase. 3 = > 15° knee hyperextension during midstance phase.	_____
C. Knee flexion moving to extension 0 = normal (knee in 4° flexion at heel strike, increasing to 15° flexion at 14% of gait cycle). 1 = normal knee flexion during early midstance phase, then knee extends to neutral. 2 = knee flexion during early midstance phase, then knee extends to full extension range (neutral or beyond) in uncontrolled manner, but not snapping back. 3 = knee in flexion during early midstance phase, then knee abruptly and forcefully extends into end range in an uncontrolled manner. D. Knee extension moving to flexion 0 = normal (knee in 4° flexion at heel strike, increasing to 15° flexion at 14% of gait cycle). 1 = knee remains in extension in early midstance, then knee flexes late, but retains control. 2 = knee remains in extension in early midstance, then knee flexes, losing control and regaining control. 3 = knee remains in extension in early midstance, then knee buckles with failure to regain control and requires use of compensatory strategies.	
14. Knee – terminal stance phase/pre-swing phase (heel-rise to toe-off) (sagittal plane) (lateral view) 0 = normal (knee flexion position in sagittal plane 35 – 45°). 1 = knee flexes < 35° or > 45°. 2 = knee flexes 35 – 45°, then extends. 3 = knee remains in full extension throughout.	_____
15. Ankle movement (sagittal plane) (lateral view). Choose __ A or __ B. (Check selection). A. Ankle plantar flexion 0 = normal (from ankle neutral position at initial heel contact, moving to 10° plantarflexion before midstance, then moving to 10° dorsiflexion at heel off). 1 = normal from initial contact (with heel strike) to midstance, but in plantarflexion after midstance. 1 = foot flat at initial contact, moving to slight plantarflexion before midstance, but in plantarflexion after midstance. 2 = foot flat at initial contact with plantarflexion to heel off. 3 = no heel contact with excessive plantarflexion to heel off. 3 = either heel contact or no heel contact followed by excessive and/or early (midstance) plantarflexion (i.e., vaulting). B. Ankle dorsiflexion 0 = normal (from ankle neutral position at initial heel contact, moving to 10° plantarflexion before midstance, then moving to 10° dorsiflexion at heel off). 1 = normal just prior to midstance, but > 10° dorsiflexion after midstance 2 = 15 – 20° dorsiflexion at midstance and to terminal stance (heel off). 3 = excessive ankle dorsiflexion (> 20°) throughout stance.	_____
16. Ankle inversion (coronal plane) (front/back view) 0 = normal (slight inversion/supination at initial stance; then eversion/pronation until heel-off). 1 = excessive ankle inversion/supination present at initial contact. 2 = excessive ankle inversion/supination present at initial contact and at midstance. 3 = excessive ankle inversion/supination throughout stance.	_____
17. Plantarflexion during terminal stance/pre-swing (heel-rise to toe-off) (sagittal plane) (lateral view) 0 = normal (adequate push-off at pre-swing for moving from dorsiflexion position to 10° plantarflexion. 1 = partial/weak push-off while moving into plantarflexion at toe-off. 2 = absent/lack of plantarflexion; no push-off.	_____
18. Toe position (sagittal plane) (lateral view) 0 = normal (toes in neutral position) 1 = excessive toe extension. 1 = clawing.	_____
**Swing Phase**	
19. Trunk posture/movement (Dynamic) (sagittal plane) (lateral view) 0 = normal (static trunk alignment maintained). 1 = trunk __ flexes or __ extends (check one) < 30°. 2 = trunk __ flexes or __ extends (check one) 30° or more.	_____
20. Trunk posture/movement (Dynamic) (coronal plane) (front/back view) 0 = normal (static trunk alignment maintained). 1 = trunk laterally flexes to __ right or to __ left (check one) < 30°. 2 = trunk laterally flexes to __ right or to __ left (check one) 30° or more.	_____
21. Pelvic position (coronal plane) (front/back view) 0 = normal (relatively level pelvis or slightly lower on swing side). 1 = mild hip hiking. 2 = moderate to severe hip hiking.	_____
22. Pelvic position (sagittal plane) (lateral view) 0 = normal (neutral position with respect to anterior or posterior tilt). 1 = anterior pelvic tilt. 1 = posterior pelvic tilt.	_____
23. Pelvic rotation as limb swings forward (transverse plane) (top view) 0 = normal (from 5° backward rotation at initiation of swing to 5° forward rotation by terminal swing) 1 = reduced pelvic rotation. 1 = excessive pelvic rotation. 2 = absent pelvic rotation.	_____
24. Hip flexion (sagittal plane) (lateral view) 0 = normal (0° hip flexion at initial swing to ~ 35° at peak, then reducing to ~ 25° at terminal swing; hip neutral with respect to hip abduction/adduction). 1 = hip begins swing in flexion, but reaches normal peak. 1 = > 10°, but < 30° hip flexion peak in the sagittal plane. 2 = > 10°, but < 30° hip flexion peak, and with hip abduction (e.g., = circumduction). 2 = > 10°, but < 30° hip flexion peak, and with hip adduction (e.g., = scissoring). 3 = 0 to 10° hip flexion throughout swing. 3 = > 35° hip flexion (excessive hip flexion).	_____
25. Hip rotation (coronal plane) (front/back view) 0 = normal (remains in neutral) 1 = abnormal, internal rotation 1 = abnormal, external rotation	_____
26. Knee – initial swing (sagittal plane) (lateral view) 0 = normal (40 – 60° of knee flexion). 1 = at least 15° knee flexion, but < 40° knee flexion. 2 = < 15° knee flexion. 3 = knee never flexes.	_____
27. Knee – midswing (sagittal plane) (lateral view) 0 = normal (60° knee flexion ± 4°). 1 = 45° - 55° knee flexion. 2 = 25° - 45° knee flexion. 3 = 0 to 25° knee flexion.	_____
28. Knee – terminal swing (sagittal plane) (lateral view) 0 = normal (from knee flexed position to full knee extension). 1 = from knee flexed position, remaining in knee flexion throughout. 1 = from knee extension position, remaining in knee extension throughout.	_____
29. Ankle movement (sagittal plane) (lateral view) 0 = normal (from initial plantarflexion at terminal stance [toe-off] to neutral by midswing, then slight dorsiflexion just prior to initial contact in stance). 1 = midswing ankle neutral but no terminal swing dorsiflexion. 2 = no midswing ankle neutral and no terminal swing dorsiflexion; plantarflexion throughout.	_____
30. Ankle inversion (coronal plane) (front/back view) 0 = normal (ankle remains in neutral regarding inversion/eversion). 1 = ankle in inverted position during swing.	_____
31. Toe position (sagittal plane) (lateral view) 0 = normal (toes in neutral position) 1 = inadequate toe extension. 1 = clawing.	_____
**Total Score _______/ 62**	
With permission: JJ Daly et al., J Neurosci Methods; 2009. 178:334-339	

**Table 2:** Directions for Administration of the Gait Assessment and Intervention Tool (G.A.I.T.).

Administration & Scoring of the Gait Assessment and Intervention Tool (G.A.I.T.) I. Preparation for video documentation of gait pattern Space- a minimum of a 10’ level walkway, with space for a camera to capture a lateral view of the entire person, head to toe, while walking. Lighting should be adequate for a clearly illuminated view of the subject. The color of the clothes should contrast with the patient/subject’s skin. First, the camera should be placed at a height of approximately mid-body level and at a location, at the mid-point of the length of the walkway for the lateral view. The lateral view video document should capture both right and left sides during walking. A second view should capture anterior/posterior (A/P), with the subject/patient walking directly toward and away from the camera. Third, a standing video document should be for a baseline posture assessment. If available, an overhead view (transverse plane) could record pelvic rotation (not used in the current publication). A minimum of 6 steps is required for analysis. If 10 feet of space does not provide the needed minimum 6 steps, use additional walkway length. The patient/subject should wear shorts or pants that can be rolled up so that at least the bottom third of his/her thighs are visible. Shirts (upper body clothing) should be tucked into the waistband to ensure viewing of the pelvic position. It is best if the patient/subject wears clothing that is well fitted, not baggy or oversized. If there is little or no (color) contrast between upper and lower body clothing, a gait belt or contrasting band or sash can be placed at the waist. Barefoot ambulation is ideal in order to assess toe position during gait. If this is not deemed safe by the evaluator then the subject/patient should wear his/her regular footwear. It can be helpful to place a piece of contrasting-color tape on each ASIS to help view pelvic movements (this was not used in the published manuscript). Physical assistance should be minimized since it can affect the patient/subject’s gait. If a person walks with the patient/subject without touching him/her, it should be noted as “stand-by assist”. Any touching of the patient/subject is considered an assist, even if the person walking with the patient/subject is loosely holding onto a gait belt. The patient/subject should ideally walk without any assistive devices and/or orthoses. If this is not deemed safe by the evaluator, then the patient/subject should use whatever devices necessary to obtain video of his/her gait. II. Instructions for scoring the Gait Assessment and Intervention Tool (G.A.I.T.) Rater training is worthwhile for greatest accuracy in any measure. For the G.A.I.T., one way to conduct rater training for one rater, is to have that rater score a patient’s gait pattern on two different days and compare their score on the two different days (intra-rater reliability). Then the rater identifies any discrepancies between their own two rating sessions, analyzes why this occurred, and corrects the thinking or action steps that led to the discrepancy. The steps for training two raters is as follows: each rater scores the same gait pattern; the two raters compare scores for each item; they discuss together how they each arrived at their score; they arrive at a consensus as to how the item should be scored for that gait pattern. If two raters are quite different in their scoring, this process should be repeated using different patients’ gait patterns until the raters are in high agreement in scoring. If more than one evaluator will be scoring the patient’s gait, be sure that each evaluator is scoring the same exact gait cycle in the taped video record. We found that stroke survivors can walk with variable gait characteristics across sequential steps in a video record. Therefore, in rater training or reliability testing of the G.A.I.T. in stroke survivors, it is critical to identify the specific gait cycle for a given patient.** View a middle step of the video record for scoring each item. The first two steps and the last two steps cannot be used for analysis/scoring because they are often affected by the acceleration and deceleration in the gait pattern. For the lateral views, whenever possible use the steps for which the camera is directly opposite the patient/subject. This ensures the best angle for scoring each item. Some items enable you to input information in addition to entering a score for the item. (for example, indicating the direction of trunk movement, or the specifics of an abnormal shoulder position). These items require a checkmark to be placed on the appropriate line in the form. For items relating to pelvic position (if overhead views are not available), view both the A/P and lateral views in order to gain insight into pelvic movement and position. If an orthotic or supportive device is worn that affects joint movement, the score for the related item would be the midpoint of the abnormal scores for said item. Example: a patient with an AFO receives a score of 2 for item #16. If an assistive device is used for ambulation (cane, walker, etc.), a normal score cannot be given for weight shifting (item 7) or for Trendelenberg (item 8). A ‘minimum abnormal’ score of 1 must be used. If the patient/subject wears shoes for the assessment and toe position cannot be evaluated, then the items pertaining to toe position should not be scored and the Total possible score adjusted. If minimal physical assistance is provided by one therapist, the scores for items pertaining to trunk alignment/posture and weight shifting should be, at a minimum, the midpoint of the abnormal scores for each item; a higher (more abnormal) score may be indicated. If, however, the physical assistance provided by one therapist appears to be moderate to extensive, or if assistance is provided by more than one therapist, the patient/subject would receive the highest abnormal score. Example: moderate assist of one or minimal assist of 2 people would warrant a score = 3 for item #4; or a score = 2 for item #5, etc.). If there is anything abnormal about the performance of the item (that is not listed), the patient/subject cannot receive a “0” (normal score) for that item. The evaluator must give a score that he/she judges appropriate based on the abnormality and the other scoring choices offered for the item. Comments pertaining to abnormalities, deviations, and/or compensations not listed on the G.A.I.T. form should be mentioned in the comment section. A total score of zero for the comprehensive form = totally normal gait (i.e., no abnormalities). The lower the overall score = the more normal the gait. It may be instructive to score both the patient/subject’s extremities for a more accurate accounting of the gait pattern. For the G.A.I.T., each side should be score separately. Specific item score instructions for each item are given in the G.A.I.T. measure. Additional tips for greater scoring accuracy of joint angles, if desired** For the items calling for assessment of degrees of motion at a given joint, the following steps can be used: 1. Place an old-fashioned transparency sheet over the video player, tv monitor screen. 2. Use the stop-frame capability to stop the video tape right at the point in the gait cycle that is being measured. 3. For example, if hip flexion is being measured, take a ruler or straight-edge and lay it along the femur between the bony landmarks used to identify the straight lines of the femur. 4. Use an erasable marker to draw on the transparency, a line between the standard bony landmarks used to identify the straight line of the femur. Then do the same for the straight line identifying pelvis/torso. 5. Place a small goniometer on the transparency and line up the arms along the femur and the pelvis/torso lines that you drew in order to obtain the value of degrees of motion for hip flexion at that given point in the gait cycle. 6. Repeat the above for the knee and ankle joints, as needed, for other GAIT items. Alternatively, if using a smartphone camera and its stop-frame capability, eliminate the transparency step and simply place a small goniometer on the phone screen, as described above, to measure the joint angle. ** new information added in response to experience and use of the measure Permission from publisher, Elsevier, to reprint with the following acknowledgement: JJ Daly et al., J Neuroscience Methods. 2009. 178:334-339. Published supplement document.

## Data Availability

Not applicable.
